# Onyx-only superior rectal artery embolisation for bleeding internal haemorrhoids: initial single-centre experience

**DOI:** 10.1186/s42155-026-00767-8

**Published:** 2026-09-07

**Authors:** Luis Loureiro, Paulo Almeida, Samuel Cardoso, Romaric Loffroy, Rui Machado

**Affiliations:** 1 Department of Angiology and Vascular Surgery, Unidade Local de Saúde de Santo António, Largo Professor Abel Salazar, 4099-001 Porto, Portugal; 2 School of Medicine and Biomedical Sciences (ICBAS), University of Porto, Porto, Portugal; 3 Grupo de Estudos Vascular, Porto, Portugal; 4 Department of Vascular and Interventional Radiology, Image-Guided Therapy Center, François-Mitterrand University Hospital, Dijon, France

**Keywords:** Haemorrhoids, Therapeutic embolisation, Rectal artery embolisation, Onyx, Rectal bleeding

## Abstract

**Purpose:**

To report an initial single-centre experience with Onyx-18-only superior rectal artery embolisation for bleeding internal haemorrhoids, focusing on technical feasibility, treatment laterality, and updated clinical outcomes.

**Materials and methods:**

Consecutive Onyx-only procedures performed between October 2023 and April 2026 in patients with bleeding-predominant internal haemorrhoids were retrospectively reviewed. Patients with portal hypertension/cirrhosis were excluded because of the distinct pathophysiology of anorectal bleeding. Patient-level clinical outcomes and procedure-level technical variables were analysed separately. Updated telephone follow-up was obtained on 12 August 2026.

**Results:**

Six patients underwent 9 procedures. Technical success was achieved in all procedures. Three patients were treated bilaterally at the initial session and 3 initially unilaterally; 2 of the latter required staged contralateral embolisation. At updated follow-up, all 6 patients reported absence of rectal bleeding after completion of treatment. Median follow-up was 19.5 months (range, 4–34), and 5 of 6 patients had at least 12 months of follow-up. One transient episode of rectal pain occurred; no major or clinically evident ischaemic complication was observed.

**Conclusion:**

Onyx-only haemorrhoidal artery embolisation was technically feasible with encouraging updated bleeding control. The observed association between bilateral treatment and fewer staged procedures is hypothesis-generating and may partly reflect an operator learning curve.

## Introduction

Haemorrhoidal artery embolisation is a minimally invasive option for bleeding-predominant internal haemorrhoids. Published series report high technical success, but clinical outcomes remain heterogeneous because of differences in patient selection, embolic agents, arterial targets, and outcome definitions [1–4]. Validated measures such as the French Bleeding Score have increasingly been used to assess durability [3, 5].

Most reported techniques use coils, particles, or combined approaches. Ethylene–vinyl alcohol copolymer (Onyx) permits slow, controlled delivery with continuous fluoroscopic visualisation of cast progression and reflux [6, 7]. These properties may facilitate distal embolisation, but Onyx is not established as a standard agent for this indication. We report an initial Onyx-18-only experience, with particular attention to treatment laterality, staged contralateral embolisation, and updated follow-up.

## Materials and methods

This single-centre retrospective service review included consecutive Onyx-18-only haemorrhoidal artery embolisations performed between October 2023 and April 2026. According to institutional policy, formal ethics committee approval and individual consent to participate were not required for this retrospective review of anonymised routine-care data. The study is reported in accordance with STROBE guidance.

Patients had bleeding-predominant internal haemorrhoidal disease after proctological assessment and colonoscopy excluding alternative luminal causes. Patients with portal hypertension/cirrhosis were excluded because portal hypertensive venous congestion represents a distinct mechanism of anorectal bleeding.

All procedures used right common femoral arterial access. After inferior mesenteric artery catheterisation, digital subtraction angiography identified superior rectal artery (SRA) branches supplying the haemorrhoidal plexus. Routine bilateral internal iliac angiography was not performed; therefore, middle rectal artery contribution was not systematically assessed and no formal SRA/middle rectal artery pattern classification was used in the revised analysis. A dimethyl-sulfoxide-compatible microcatheter was advanced superselectively and Onyx-18 was injected slowly under continuous fluoroscopy without adjunctive coils or particles. Bilateral embolisation was defined as treatment of both right- and left-sided SRA territories during the same session. Staged contralateral embolisation was performed when persistent or recurrent bleeding was associated with untreated contralateral SRA supply.

Technical success was completion of the intended Onyx-18 embolisation with satisfactory target devascularisation. The principal clinical endpoint was absence of rectal bleeding at the latest follow-up. Earlier rebleeding, repeat embolisation, additional haemorrhoid-directed treatment, and adverse events were also recorded. Updated telephone follow-up on 12 August 2026 documented bleeding status and any further intervention. Formal French Bleeding Score, quality-of-life, and validated satisfaction instruments had not been collected prospectively. No patient had previously undergone Doppler-guided haemorrhoidal artery ligation or transanal haemorrhoidal dearterialisation.

Analysis was descriptive. Patient-level outcomes and procedure-level technical variables were kept separate. ChatGPT (OpenAI) was used for language editing, structural refinement, and formatting assistance; all data, analyses, references, interpretations, and final wording were independently verified and approved by the authors.

## Results

The cohort comprised 6 patients (median age, 43 years; range, 24–80; 5 men) who underwent 9 Onyx-only procedures. Baseline Goligher grades were I in 1 patient, II in 2, III in 2, and IV in 1. All 6 reported bleeding several times per week or daily. Two had received iron therapy and 1 had required red blood cell transfusion before embolisation. Three patients had received previous haemorrhoid-directed treatment, including sclerotherapy, rubber-band ligation, or surgery; none had undergone haemorrhoidal artery ligation/dearterialisation (Table 1).

**Table 1:** Patient characteristics and clinical outcomes

Characteristic	Value
Patients, *n*	6
Age, median (range), years	43 (24–80)
Male sex, *n*	5
Goligher grade I/II/III/IV, *n*	1/2/2/1
Previous haemorrhoid-directed treatment, *n*	3
Previous HAL/THD, *n*	0
Initial unilateral/bilateral treatment, *n*	3/3
Staged contralateral embolisation, *n*	2
Absence of rectal bleeding at latest follow-up, *n*	6
Follow-up, median (range), months	19.5 (4–34)
Follow-up ≥ 12 months, *n*	5
Complementary surgery for residual/prolapsing pedicles, *n*	3

*HAL*, haemorrhoidal artery ligation; *THD*, transanal haemorrhoidal dearterialisation

Technical success was achieved in all 9 procedures. Six procedures were unilateral and 3 bilateral. Initial treatment was unilateral in 3 patients and bilateral in 3. Two of the 3 patients initially treated unilaterally required staged contralateral embolisation; none of the 3 initially treated bilaterally required repeat embolisation. This temporal shift from unilateral to bilateral treatment occurred as operator experience increased and therefore cannot be interpreted as a comparison of treatment strategies. Median procedure time was 38 min (range, 28–53) and median radiation dose was 838 mGy (range, 267–2026). Onyx volume was documented in 6 of 9 procedures (median, 1.6 mL; range, 0.3–4.0); volumes were unavailable for 3 early procedures because they were not recorded in the procedural reports. One transient episode of rectal pain occurred after bilateral embolisation. No major adverse event or clinically evident non-target or ischaemic complication was recorded (Table 2).

**Table 2:** Procedure-level characteristics

Characteristic	Value
Procedures, *n*	9
Technical success, *n*	9
Unilateral/bilateral procedures, *n*	6/3
Procedure time, median (range), min	38 (28–53)
Radiation dose, median (range), mGy	838 (267–2026)
Onyx volume documented, procedures	6/9
Onyx volume, median (range), mL*	1.6 (0.3–4.0)
Progreat 2.0 Fr/Progreat 2.7 Fr/ASAHI Veloute 1.7 Fr, *n*	5/1/3
Routine internal iliac angiography, *n*	0
Minor/major adverse events, *n*	1/0

^*^Onyx volume was not documented in the procedural reports for 3 early procedures; missing values were not interpreted as zero. All procedures used right common femoral access and Onyx-18 without adjunctive coils or particles. Middle rectal artery contribution was not systematically assessed because routine internal iliac angiography was not performed. The only minor event was transient rectal pain

**Fig. 1:**
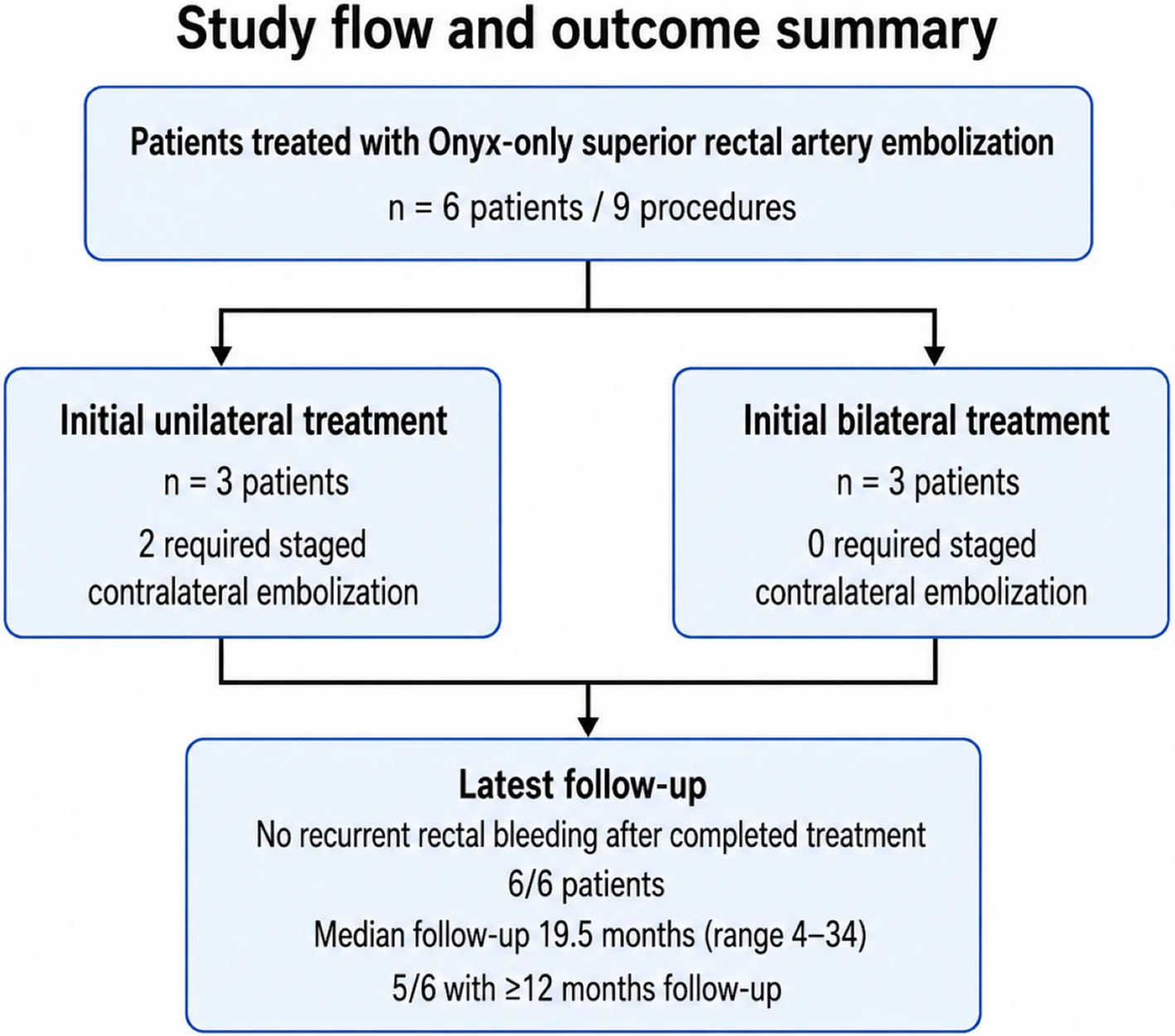
Study flow and outcome summary. Six patients underwent 9 Onyx-18-only superior rectal artery embolisation procedures. Initial treatment was unilateral in 3 patients and bilateral in 3; 2 patients initially treated unilaterally required staged contralateral embolisation, whereas none initially treated bilaterally did. All 6 patients reported absence of recurrent rectal bleeding after completion of treatment at latest follow-up; median follow-up was 19.5 months (range, 4–34), with at least 12 months in 5 of 6 patients

**Fig. 2:**
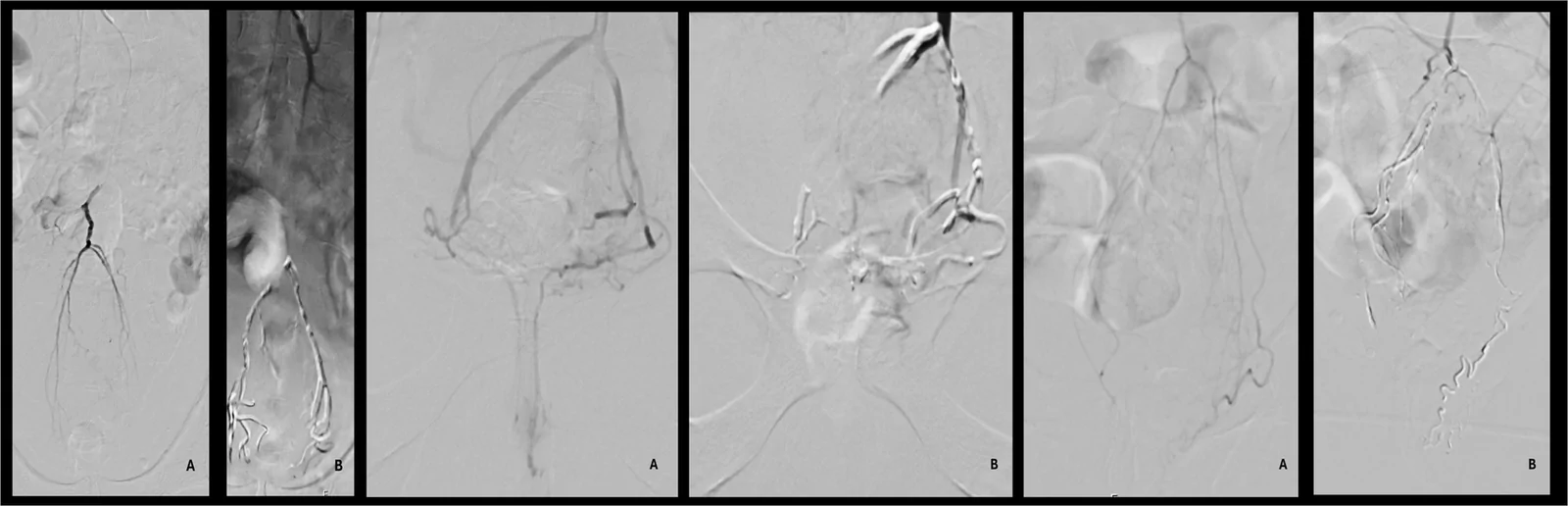
Bilateral Onyx-only superior rectal artery embolisation in three representative patients. **A** Pre-intervention selective angiography demonstrating bilateral superior rectal artery branches supplying the haemorrhoidal plexus. **B** Final post-embolisation images demonstrating distal target devascularisation and radiopaque Onyx-18 cast formation within the treated superior rectal artery branches

At updated follow-up, all 6 patients reported absence of rectal bleeding after completion of treatment. Median follow-up from the first embolisation was 19.5 months (range, 4–34), with at least 12 months of follow-up in 5 of 6 patients. Three patients with baseline Goligher grade III–IV subsequently underwent complementary surgery for residual or prolapsing pedicles after bleeding control; these operations were not performed for recurrent bleeding (Figs. 1 and 2).

## Discussion

This short series supports the technical feasibility of Onyx-18-only SRA embolisation and provides updated clinical follow-up beyond 12 months in 5 of 6 patients. All 6 were free from rectal bleeding at the latest contact after completion of treatment. These findings should be interpreted as preliminary: recent evidence demonstrates that haemorrhoidal embolisation outcomes may decline over time, and validated bleeding scores are important for durable outcome assessment [3, 5].

Onyx provides directly visible, progressive distal embolisation and allows delivery to be paused or resumed [6, 7]. These characteristics are attractive in small, tortuous SRA branches, although any theoretical advantage over particles or coils remains unproven. The absence of major or clinically evident ischaemic complications in this small series does not establish safety; serious ischaemic events after haemorrhoidal embolisation have been reported, and selective delivery with strict control of reflux remains essential [8].

Two of 3 patients initially treated unilaterally required staged contralateral embolisation, whereas none of 3 initially treated bilaterally did. This is biologically plausible because untreated contralateral SRA inflow may maintain the bleeding plexus. However, later cases were more often treated bilaterally, reflecting increasing operator confidence and a learning curve. The study therefore does not demonstrate superiority of bilateral embolisation. It only suggests that, when both SRA territories can be safely and selectively catheterised, complete bilateral target treatment may reduce the need for a staged procedure. Haemorrhoidal artery ligation/dearterialisation is the closest surgical analogue because it is based on the same arterial-inflow concept [9]; none of our patients had previously undergone this technique.

Limitations include the retrospective design, very small cohort, absence of a comparator, heterogeneous follow-up, lack of prospectively collected French Bleeding Score, quality-of-life or validated satisfaction measures, and missing Onyx-volume documentation in 3 early procedures. Routine internal iliac angiography was not performed, so middle rectal artery supply was not systematically assessed. The findings may not generalise beyond carefully selected patients with bleeding-predominant internal haemorrhoids. These limitations preclude comparison with established surgical techniques or inference regarding long-term superiority. Prospective studies using validated bleeding scores and standardised angiographic assessment are warranted.

## Conclusion

Onyx-18-only superior rectal artery embolisation was technically feasible and provided encouraging bleeding control in this small, selected cohort. Bilateral treatment may reduce staged contralateral embolisation, but this signal is confounded by operator learning and requires prospective confirmation.

## Data Availability

The datasets generated and/or analysed during the current study are not publicly available because of patient privacy and institutional data-protection requirements, but anonymised data are available from the corresponding author on reasonable request and subject to institutional approval.

## Abbreviations

DMSO: Dimethyl sulfoxide
RBC: Red blood cell
SRA: Superior rectal artery
STROBE: Strengthening the Reporting of Observational Studies in Epidemiology

## References

[1] Panneau J, Mege D, Di Bisceglie M, Duclos J, Habert P, Bartoli A, et al. Rectal artery embolization for hemorrhoidal disease: anatomy, evaluation, and treatment techniques. Radiographics. 2022;42(6):1829–44. 10.1148/rg.220014. 36190848

[2] De Gregorio MA, Guirola JA, Serrano-Casorran C, Gutierrez C, Gregorio A, Sierre S, et al. Catheter-directed hemorrhoidal embolization for rectal bleeding due to hemorrhoids (Goligher grade I-III): prospective outcomes from a Spanish emborrhoid registry. Eur Radiol. 2023;33(12):8754–63. 10.1007/s00330-023-09923-3. 37458757

[3] Nguyenhuy M, Xu Y, Kok HK, Maingard J, Joglekar S, Jhamb A, et al. Clinical outcomes following rectal artery embolisation for the treatment of internal haemorrhoids: a systematic review and meta-analysis. Cardiovasc Intervent Radiol. 2022;45:1351–61. 10.1007/s00270-022-03154-7. 35551442

[4] Golezar MH, Ghorani H, Alemi F, Fayedeh F, Yeganegi M, Toutounchian S, et al. Arterial embolization for the internal hemorrhoids management: a systematic review. Health Sci Rep. 2026;9:e71577. 10.1002/hsr2.71577. 41503542 PMC12772977

[5] Cai H, Zhang H, Pang R, Zhang M, Wang W, Yin L, et al. Short- and medium-term efficacy of super-selective haemorrhoidal artery embolisation for internal haemorrhoids and its relationship with severity grading: a single-centre retrospective study. J Med Imaging Radiat Oncol. 2026. 10.1111/1754-9485.70165. 42573173

[6] Ne R, Chevallier O, Falvo N, Facy O, Berthod PE, Galland C, et al. Embolization with ethylene vinyl alcohol copolymer (Onyx) for peripheral hemostatic and non-hemostatic applications: a feasibility and safety study. Quant Imaging Med Surg. 2018;8(3):280–90. 10.21037/qims.2018.04.03. 29774181 PMC5941214

[7] Urbano J, Cabrera JM, Franco A, Alonso-Burgos A. Selective arterial embolization with ethylene-vinyl alcohol copolymer for control of massive lower gastrointestinal bleeding: feasibility and initial experience. J Vasc Interv Radiol. 2014;25(6):839–46. 10.1016/j.jvir.2014.02.024. 24755085

[8] Hess TN, Baron JJ. Colonic ischemia and necrosis following hemorrhoid embolization: a brief report. Am Surg. 2026;92(1):291–2. 10.1177/00031348251378901. 40921478

[9] Ratto C, Campenni P, Papeo F, Donisi L, Litta F, Parello A. Transanal hemorrhoidal dearterialization (THD) for hemorrhoidal disease: a single-center study on 1000 consecutive cases and a review of the literature. Tech Coloproctol. 2017;21(12):953–62. 10.1007/s10151-017-1726-5. 29170839 PMC5830492

